# Design Method for a Higher Order Extended Kalman Filter Based on Maximum Correlation Entropy and a Taylor Network System

**DOI:** 10.3390/s21175864

**Published:** 2021-08-31

**Authors:** Qiupeng Wang, Xiaohui Sun, Chenglin Wen

**Affiliations:** 1 School of HDU-ITMO, Joint Institute, Hangzhou Dianzi University, Hangzhou 310018, China; wangqp@hdu.edu.cn; 2 School of Automation, Hangzhou Dianzi University, Hangzhou 310018, China; sun_xh1993@hdu.edu.cn; 3 School of Automation, Guangdong University of Petrochemical Technology, Maoming 525000, China

**Keywords:** pseudolinearization, maximum entropy Kalman filter, multidimensional Taylor network

## Abstract

This paper proposes one new design method for a higher order extended Kalman filter based on combining maximum correlation entropy with a Taylor network system to create a nonlinear random dynamic system with modeling errors and unknown statistical properties. Firstly, the transfer function and measurement function are transformed into a nonlinear random dynamic model with a polynomial form via system identification through the multidimensional Taylor network. Secondly, the higher order polynomials in the transformed state model and measurement model are defined as implicit variables of the system. At the same time, the state model and the measurement model are equivalent to the pseudolinear model based on the combination of the original variable and the hidden variable. Thirdly, higher order hidden variables are treated as additive parameters of the system; then, we establish an extended dimensional linear state model and a measurement model combining state and parameters via the previously used random dynamic model. Finally, as we only know the results of the limited sampling of the random modeling error, we use the combination of the maximum correlation estimator and the Kalman filter to establish a new higher order extended Kalman filter. The effectiveness of the new filter is verified by digital simulation.

## 1. Introduction

The application of filters occupies an important position in various fields at the national and international levels. The progress and development of filters play important roles in national economic construction—especially national defense construction—such as in real-time estimation and target tracking. In 1960, Kalman proposed a method of filtering under the minimum mean squared error criterion for linear systems, and it soon began to be widely used [1]. In order to solve nonlinear problems, extended Kalman filters (EKFs) [2], unscented Kalman filters (UKFs) [3], and cubature Kalman filters (CKFs) have since emerged. However, the above-mentioned filtering methods require the modeling error to be Gaussian white noise. As such, their performances are likely to worsen when applied to non-Gaussian situations, especially when the systems are disturbed by impulsive noise. Impulsive noise arises from heavy-tailed distributions [4] (such as some mixed Gaussian distributions), and is common in many real scenarios of automatic control and target tracking (for instance, the measurement noise in the radar system is often not Gaussian noise, but heavy-tailed non-Gaussian noise [5]). In 1993, Gordon and Salmond proposed particle filtering when the density function is known [6]; this achieves an approximation of the distribution function by sampling a large number of particles therein. However, this method is very complicated; it requires a large number of particles, and it will cause particle degradation after re-sampling. In general, the density function is difficult to obtain. For this reason, for the linear system, Chen designed the corresponding Kalman filter under the maximum correlation entropy criterion based on the limited realization of random variables [7]; this is called the maximum correlation entropy Kalman filter (MCKF) [8]. On this basis, the maximum correntropy extended Kalman filter (MCEKF) and the maximum correntropy unscented Kalman filter (MCUKF), which can solve nonlinear non-Gaussian systems, have since emerged [9]. However, in MCEKFs, all higher order terms in the Taylor expansion are discarded. Therefore, a large truncation error will be generated, and the filtering performance will decrease or even diverge as the nonlinearity of the system increases. In addition, each step of the state estimation needs to recalculate the Taylor expansion coefficient, which will undoubtedly increase the complexity of the calculation. MCUKFs use UT transformation and sigma point sampling [10]; this is called deterministic sampling. There is only one sampling point for a dimensional system. Neither low-dimensional nor high-dimensional systems have a strong claim to superiority. A large number of experiments have shown that both EKFs and UKFs can be approximated by a second-order polynomial at most [11], which will produce a large rounding error. Hence, both will eventually face the problems of degraded filtering performance and divergence as their nonlinearity increases [12].

This project proposes a higher order extended Kalman filter method based on maximum correlation entropy, under the assumption that both state and measurement equations can be modeled and based on a strong nonlinear function. The main contributions of this paper are as follows: (1) using multidimensional Taylor nets to convert the general expression of nonlinear functions into higher order polynomials; (2) defining each order of polynomial in the system as hidden variables of the corresponding order, and treating them as time-variable parameters; (3) establishing the dynamic relationship between the time-variable parameters and combining them with the original variables to further establish the expanded dimension state model; (4) based on the expanded linear state variables, equivalently rewriting the measurement model into the corresponding linear form; and (5) according to the established linear state and measurement model of the new extended dimension system, establishing a higher order extended Kalman filter method based on maximum correlation entropy.

The remaining parts of this paper are organized as follows: the first chapter is the preface of our knowledge, which introduces the definition of “entropy”; the Section 2 presents a method for identifying nonlinear functions based on multidimensional Taylor networks; the Section 3 presents a higher order extended Kalman filter method; the Section 4 presents the detailed design process of the maximum correlation entropy higher order extended Kalman filter; the Section 5 concerns simulation verification; and the Section 6 and Section 7 presents a summary and outlook.

## 2. Description of Correntropy

Correntropy is a generalized similarity measure between two random variables [13]. Given two one-dimensional random variables $\varphi ,\zeta \in R^{1}$ , their joint distribution function is $F_{\phi \xi }(\varphi ,\zeta )$ ; then, the correlation entropy is defined as follows:

(1) $$V(\phi ,\xi )=\varepsilon \left[\alpha (\varphi ,\zeta )\right]=\int \alpha (\varphi ,\zeta )dF_{\phi \xi }(\varphi ,\zeta )$$

where ε is the expectation operator and α(⋅,⋅) is the translation-invariant Mercer kernel. In this article, it is not particularly emphasized that this kernel function is a Gaussian kernel, which is defined as follows:

(2) $$\alpha \left(\varphi ,\zeta \right)=G_{\tau }(e)=exp\left(-\frac{e^{2}}{2\tau^{2}}\right)$$

where e=φ−ζ , τ>0 represents the kernel’s bandwidth.

By expanding Equation (2) with a Taylor series, we can obtain the following:

(3) $$\alpha (\varphi ,\zeta )=G_{\tau }(e)=exp\left(-\frac{e^{2}}{2\tau^{2}}\right)=\sum_{k=0}^{\infty }\frac{{\left(-1\right)}^{k}}{2^{k}\tau^{2k}k!}\varepsilon \left\{{\left(\varphi -\zeta \right)}^{2k}\right\}$$

and then the correlation entropy of Equation (1) has the following expression:

(4) $$\begin{array}{l} V(\phi ,\xi )=\varepsilon [\alpha (\varphi ,\zeta )]=\int \sum_{k=0}^{\infty }\frac{{\left(-1\right)}^{k}}{2^{k}\tau^{2k}k!}{\left(\varphi -\zeta \right)}^{2k}dF_{\phi \xi }(\varphi ,\zeta )\\ \;\;\;\;\;=\sum_{k=0}^{\infty }\frac{{\left(-1\right)}^{k}}{2^{k}\tau^{2k}k!}\int {\left(\varphi -\zeta \right)}^{2k}dF_{\phi \xi }(\varphi ,\zeta )=\sum_{k=0}^{\infty }\frac{{\left(-1\right)}^{k}}{2^{k}\tau^{2k}k!}\varepsilon \left\{{\left(\phi -\xi \right)}^{2k}\right\} \end{array}$$

where $\varepsilon \left\{{\left(\phi -\xi \right)}^{2k}\right\}=\int^{\;}{\left(\varphi -\zeta \right)}^{2k}dF_{\phi \xi }\left(\varphi ,\zeta \right)$ is the 2k truncation statistic of the random variable ϕ,ξ∈R .

However, in most practical cases, joint distribution $F_{\phi \xi }$ is usually unknown, and there are often finite implementations $\left(\varphi^{\left(j\right)},\zeta^{\left(j\right)}\right),j=1,2,\cdots ,N$ of (ϕ,ξ) for random variables. In these cases, the sample mean estimator can be used to estimate the heterogeneity:

(5) $$\varepsilon \left\{{\left(\phi -\xi \right)}^{2k}\right\}=\frac{1}{N}\left(\sum_{j=0}^{N}{\left(\varphi^{\left(j\right)}-\zeta^{\left(j\right)}\right)}^{2k}\right)$$

Then, the entropy expression of the random variable pair  (φ, ζ) is driven by finite data:

(6) $$\begin{array}{l} \hat{V}(\phi ,\xi )=\varepsilon [\alpha (\varphi ,\zeta )]\\ \;\;\;\;\;=\int \sum_{k=0}^{\infty }\frac{{\left(-1\right)}^{k}}{2^{k}\tau^{2k}k!}{\left(\varphi -\zeta \right)}^{2k}dF_{\phi \xi }(\varphi ,\zeta )\\ \;\;\;\;\;=\sum_{k=0}^{\infty }\frac{{\left(-1\right)}^{k}}{2^{k}\tau^{2k}k!}\frac{1}{N}\left(\sum_{j=0}^{N}{\left(\varphi^{\left(j\right)}-\zeta^{\left(j\right)}\right)}^{2k}\right)\\ \;\;\;\;\;=\frac{1}{N}\sum_{k=0}^{\infty }\frac{{\left(-1\right)}^{k}}{2^{k}\tau^{2k}k!}\left(\sum_{j=0}^{N}{\left(\varphi^{\left(j\right)}-\zeta^{\left(j\right)}\right)}^{2k}\right)\\ \;\;\;\;\;=\frac{1}{N}\sum_{k=0}^{\infty }\frac{{\left(-1\right)}^{k}}{2^{k}\tau^{2k}k!}\left(\sum_{j=0}^{N}{\left(e^{\left(j\right)}\right)}^{2k}\right)=\frac{1}{N}\sum_{j=0}^{N}G_{\tau }\left(e^{\left(j\right)}\right) \end{array}$$

When $\phi ,\xi \in R^{n}$ , and the components of vector e=ϕ−ξ are independent of one another, multidimensional correlation entropy is based on N sampling.

## 3. Non-Linear Model Identification Based on Multidimensional Taylor Networks

Given that the state model and observation model are complex dynamic systems with nonlinear characteristics [14]:

(7) α(τ+1)=σ(α(τ))+γ(τ)

(8) Γ(τ+1)=δ(α(τ+1))+θ(τ+1)

where $\alpha (\tau )\in R^{h}$ is an h-dimensional state vector; $T(\tau +1)\in R^{d}$ represents the d-dimensional measure vector; and $\sigma_{i}(\alpha (\tau )),\;i=1,2,\cdots ,h$ and $\delta_{j}(\alpha (\tau +1)),\;j=1,2,\cdots ,d$ represent the state function and the measurement function, respectively. The modeling errors for non-Gaussian systems are γ(k) and θ(k) , while $\vartheta =diag\{\vartheta_{1},\vartheta_{2},\cdots ,\vartheta_{h}\}$ and $\eta =diag\left(\begin{array}{cccc} \eta_{1} & \eta_{2} & \cdots & \eta_{d} \end{array}\right)$ are the process noise variance and the measurement noise variance, respectively.

**Lemma** **1.** *Any continuous function defined in a closed interval can be approximated accurately with a polynomial function* [15].

**Lemma** **2.** *For continuous functions,* σ(α(k)) *, defined in a closed interval, can be approximated by the following* [16]:

(9) $$\sum_{i=1}^{N(h,l)}\psi_{i}(k)\prod_{t=1}^{l}\alpha_{t}^{\lambda_{i,t}}(k)$$

*where* N(h,l) *denotes the total number of terms in the expansion and* $\lambda_{i,t}$ *denotes the power of the variable* $\alpha_{t}$ *in the product of the ith variable.*

### 3.1. Multidimensional Taylor Network Structure

The multidimensional Taylor network model can replace the traditional neural network with the dynamic model and control the system under certain conditions; it is characterized by a nonlinear autoregressive moving-average model composed of polynomials. The multidimensional Taylor network (MTN) uses a forward single intermediate layer structure, including an input layer, an intermediate layer, and an output layer. Supposing that the input layer comprises n nodes— $\alpha (\tau )={\left[\begin{array}{cccc} \alpha_{1}(\tau ) & \alpha_{2}(\tau ) & \cdots & \alpha_{h}(\tau ) \end{array}\right]}^{T}\in R^{h}$ —the output layer is α(τ+1) , the middle layer is the network processing layer, and each input variable realizes the weighted summation of each power product term in this layer. The middle layer is composed of various power product terms and the corresponding connection weight vector $\psi_{j}(\tau )$ :

$$\psi_{j}(\tau )={\left[\psi_{j,1}(\tau ),\psi_{j,2}(\tau ),\cdots ,\psi_{j,N(h,l)}(\tau )\right]}^{T}$$

which represents the output weight vector connecting the intermediate layer and the output node of the network.

According to the multivariate Taylor equation, if a function is differentiable to the h+1 th order at a certain point, then the function expands to a form where the power series of the variable is not greater than m times. The model can be expressed as a dynamic equation, as follows:

(10) $$\alpha_{j}(\tau +1)=\sigma (\alpha (\tau ))=\sum_{i=1}^{N(h,l)}\psi_{j,i}(\tau )\prod_{t=1}^{l}\alpha_{t}^{\lambda_{i,t}}(\tau )+\Delta \sigma (\tau )$$

where σ(⋅) is a function of nonlinearity described by a multidimensional Taylor network model, $\psi_{i}$ represents the weight before the product item of the ith variable, N(h,l) denotes the total number of terms in the expansion, $\lambda_{i,t}$ denotes the power of the variable $\alpha_{t}$ in the product of the ith variable, and Δσ(τ) is the error—also known as the remainder—produced by the identification of a function by a multidimensional Taylor network.

### 3.2. Parameter Identification Method Based on Kalman Filtering

#### Model Establishment of a Kalman Filter

A Kalman filter can be regarded as an optimized autoregressive data processing method that describes the entire system through a state equation and an observation equation.

State equation:

(11) $$\psi_{j,i}(\tau +1)=\psi_{j,i}(\tau )+w_{j,i}(\tau )$$

where i=1,2,⋯,N(h,l) , j=1,2⋯,h .

**Figure 1:**
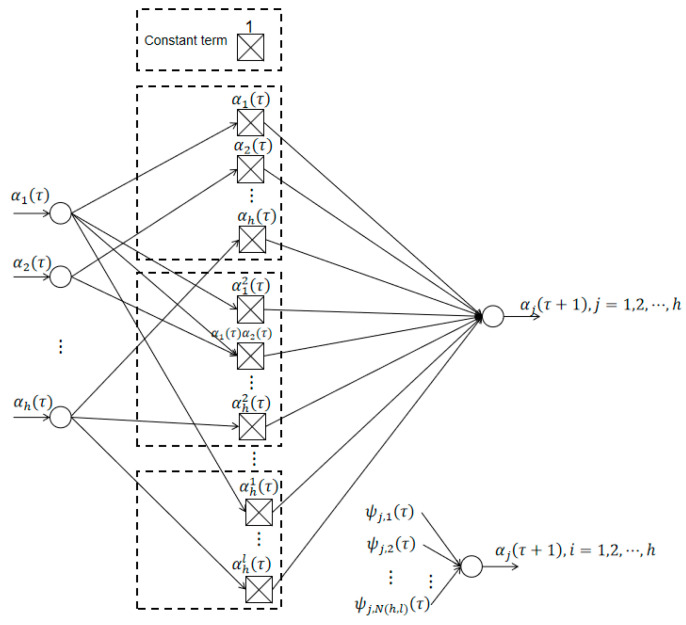
Model of a multidimensional Taylor network.

Observation equation:

It is not difficult to draw from Figure 1:

$$\begin{array}{l} \alpha_{j}(\tau +1)=\sum_{i=1}^{N(h,l)}\psi_{j,i}(\tau +1)\prod_{t=i}^{l}\alpha_{t}^{\lambda_{i,t}}(\tau +1)\\ \;\;\;\;\;=H_{j}(\tau +1)\cdot {\left[\psi_{j,1}(\tau +1),\psi_{j,2}(\tau +1),\cdots ,\psi_{j,N(h,l)}(\tau +1)\right]}^{T}+v_{j}(\tau +1)\\ \;\;\;\;\;=H_{j}(\tau +1)\cdot \psi_{j}(\tau +1)+v_{j}(\tau +1) \end{array}$$

Thus,

(12) $$\begin{array}{l} \alpha (\tau +1)={\left[\alpha_{1}(\tau +1),\alpha_{2}(\tau +1),\cdots ,\alpha_{j}(\tau +1),\cdots ,\alpha_{h}(\tau +1)\right]}^{T}\\ \;\;\;\;=H(\tau )\cdot \psi (\tau +1)+v(\tau +1) \end{array}$$

where $H(\tau )={\left[H_{1}(\tau +1),H_{2}(\tau +1),\cdots ,H_{h}(\tau +1)\right]}^{T}$ ; $\psi (\tau +1)=\left[\psi_{1}(\tau +1),\psi_{2}(\tau +1),\cdots ,\psi_{h}(\tau +1)\right]$ ; $\psi_{j,i}(\tau +1)$ represents the system state at τ -time, that is, the parameter status value of the kth moment; and β(τ+1) represents the output value of the network. It is assumed that both process noise w(τ) and v(τ+1) are Gaussian white noise during the analysis, and $Q_{j}=diag\left(\begin{array}{cccc} Q_{j,1} & Q_{j,2} & \cdots & Q_{j,N(h,l)} \end{array}\right)$ and $R_{j}=diag\left(\begin{array}{cccc} R_{j,1} & R_{j,2} & \cdots & R_{j,N(h,l)} \end{array}\right)$ , which are the process noise variance and measurement noise variance, respectively. Here, we use a Kalman filter to approximate the dynamic model. As the filtering principle of Kalman filters is mentioned later in this article, please refer to Equations (20)–(24) for the detailed process.

### 3.3. Approximation Analysis

Given a class of nonlinear functions σ(α(k)) , it can be assumed that it is derivative of the rth order, but r is a relatively large number, making it difficult for us to use Taylor nets to approximate its function. The optimal approach would be to set m,1≤m≤r and use the Taylor network to expand the nonlinear function to the mth order, obtain the result of Equation (16), and simultaneously ensure the higher order error term Δδ≤θ , where θ is the acceptable error threshold. This not only makes the Taylor network fitting function process easier, but also ensures the accuracy of the fit.

## 4. Higher Order Extended Kalman Filter

### 4.1. Pseudolinearized Representation of Nonlinear Functions

For ease of description and understanding, if l=d=2 , we can expand Equation (7) through a multidimensional Taylor network to the mth order, as follows:

(13) $$\begin{array}{l} \sigma_{i}(\alpha (k))=\left(\omega_{i,1,0}\alpha_{1}(\tau )+\omega_{i,0,1}\alpha_{2}(\tau )\right)\\ \begin{array}{l} \;\;\;\;\;\;\;+\left(\omega_{i,2,0}\alpha_{1}{\left(\tau \right)}^{2}+\omega_{i,1,1}\alpha_{1}(\tau )\alpha_{2}(\tau )+\omega_{i,0,2}\alpha_{2}{\left(\tau \right)}^{2}\right)\\ \;\;\;\;\;\;\;+\left(\omega_{i,3,0}\alpha_{1}{\left(\tau \right)}^{3}+\omega_{i,1,2}\alpha_{1}(\tau )\alpha_{2}^{2}(\tau )+\omega_{i,2,1}\alpha_{1}^{2}(\tau )\alpha_{2}(\tau )+\omega_{i,0,3}\alpha_{2}{\left(\tau \right)}^{3}\right)\\ \;\;\;\;\;\;\;+\sum_{\begin{array}{c} l_{1}+l_{2}=l\\ l_{1}+l_{2}\leq l \end{array}}\omega_{i,l_{1},l_{2}}\alpha_{1}^{l_{1}}(\tau )\alpha_{2}^{l_{2}}(\tau )+\cdots +\sum_{\begin{array}{c} m_{1}+m_{2}=r\\ m_{1}+m_{2}\leq r \end{array}}\omega_{i,r_{1},r_{2}}\alpha_{1}^{m_{1}}(\tau )\alpha_{2}^{m_{2}}(\tau )+\Delta \sigma_{i}(\tau ) \end{array} \end{array}$$

where $\sum_{\begin{array}{l} l_{1}+l_{2}=l\\ l_{1},l_{2}\leq l \end{array}}\alpha_{1}^{l_{1}}(\tau )\alpha_{2}^{l_{2}}(\tau )$ is the sum of all tensors of the l th order and $\omega_{i,l_{1},l_{2}}$ represents the weight corresponding to each order of the tensor.

**Definition** **1.**
$\alpha^{\left(l\right)}(\tau )=\{\alpha_{1}^{l_{1}}(\tau )\alpha_{2}^{l_{2}}(\tau )\cdots \alpha_{2}^{l_{h}}(\tau ),\;\;\;l_{1}+l_{2}+l_{h}=l;\;\;\;0\leq l_{j}\leq l;\;\;l=0,1,\cdots ,h\}$ *is a set of implicit variables of the*  l  *th order.*

**Definition** **2.**
$\omega_{i}^{\left(l\right)}=\left[\omega_{i;\;1}^{\left(l\right)},\;\omega_{i;\;2}^{\left(l\right)},\;\cdots ,\omega_{i;\;n_{l}}^{\left(l\right)}\right]=\left[\omega_{i;\;l,0},\;\omega_{i;\;l-1,1},\cdots ,\;\omega_{i;\;0,l}\right],\;\;\;i=1,2,\cdots l$ *is the weight vector corresponding to the ith order implicit variable.*

In [17], there is a detailed pseudolinearization process, so we will not repeat it in this article. In order to make the model more accurate, we treat the remainder Δσ(τ) of the equation of state as latent variables. According to Definition 1 and Definition 2, the pseudolinear extended dimension form using the remainder as a hidden variable is as follows:

(14) $$\alpha^{\left(1\right)}(\tau +1)=W^{\left(1\right)}(\tau +1,\tau )\alpha^{\left(1\right)}(\tau )+\sum_{l=2}^{m}W^{\left(l\right)}(\tau +1,\tau )\alpha^{\left(l\right)}(\tau )+C\cdot \Delta \sigma (\tau )+\gamma^{\left(1\right)}(\tau )$$

where $\alpha^{\left(1\right)}(\tau )=\left[\begin{array}{c} \alpha_{1}^{\left(1\right)}(\tau )\\ \alpha_{2}^{\left(1\right)}(\tau ) \end{array}\right]$ , $W^{\left(l\right)}=\left[\begin{array}{c} \omega_{1}^{\left(l\right)}\\ \omega_{2}^{\left(l\right)} \end{array}\right]$ , $\gamma (\tau )=\gamma^{\left(1\right)}(\tau )=\left[\begin{array}{c} \gamma_{1}^{\left(1\right)}(\tau )\\ \gamma_{2}^{\left(1\right)}(\tau ) \end{array}\right]$ , $C=\left[\begin{array}{cc} 1 & 0\\ 0 & 1 \end{array}\right]$ .

Similarly, Equation (8) can be rewritten as follows:

(15) $$\Gamma^{\left(1\right)}(\tau +1)=\chi^{\left(1\right)}(\tau +1)\alpha^{\left(1\right)}(\tau +1)+\sum_{l=2}^{m}\chi^{\left(l\right)}(\tau +1)\alpha^{\left(l\right)}(\tau +1)+D\cdot \Delta \sigma (\tau +1)+\theta^{\left(1\right)}(\tau +1)\;$$

where $\Gamma^{\left(1\right)}(\tau +1)=\left[\begin{array}{c} \Gamma_{1}^{\left(1\right)}(\tau )\\ \Gamma_{2}^{\left(1\right)}(\tau ) \end{array}\right]$ , $\chi^{\left(l\right)}=\left[\begin{array}{c} \chi_{1}^{\left(l\right)}\\ \chi_{2}^{\left(l\right)} \end{array}\right]$ , $\theta^{\left(1\right)}(\tau +1)=\left[\begin{array}{c} \theta_{1}^{\left(1\right)}(\tau +1)\\ \theta_{2}^{\left(1\right)}(\tau +1) \end{array}\right]$ , $D=\left[\begin{array}{cc} 0 & 0\\ 0 & 0 \end{array}\right]$ .

### 4.2. Linearized Representation of Nonlinear Functions

In order to transform the pseudolinear model established in Section 3.1 into a true linear form, it is necessary to establish a dynamic relationship between the lth order hidden variables and the uth order hidden variables [18]:

(16) $$\alpha^{\left(l\right)}(\tau +1)=W_{l}^{\left(u\right)}(\tau )\alpha^{\left(u\right)}(\tau )\;\;l,u=2,3,\cdots ,m$$

where W can be identified based on the multidimensional Taylor network in its original state; without any prior information, it can be set as follows:

(17) $$W_{l}^{\left(u\right)}(\tau )=\{\begin{array}{l} I,\;\;\;l=u\\ 0,\;\;\;l\neq u \end{array}$$

Combining Definition 1, Definition 2, and Equation (19), the state model Equation (7) has the following linear matrix form:

If $A(\tau )={\left[{\left(\alpha^{\left(1\right)}(\tau )\right)}^{T},\;{\left(\alpha^{\left(2\right)}(\tau )\right)}^{T},\;\cdots \;,\;{\left(\alpha^{\left(l\right)}(\tau )\right)}^{T},\;\cdots \;,\;{\left(\alpha^{\left(r\right)}(\tau )\right)}^{T},\Delta \sigma (\tau )\right]}^{T}$

$$W(\tau +1,\tau )=\left[\begin{array}{cccccccc} W_{1}^{\left(1\right)}(\tau ) & W_{1}^{\left(2\right)}(\tau ) & \cdots & W_{1}^{\left(u\right)}(\tau ) & \cdots & W_{1}^{\left(m-1\right)}(\tau ) & W_{1}^{\left(m\right)}(\tau ) & C\\ W_{2}^{\left(1\right)}(\tau ) & W_{2}^{\left(2\right)}(\tau ) & \cdots & W_{2}^{\left(u\right)}(\tau ) & \cdots & W_{2}^{\left(m-1\right)}(\tau ) & W_{2}^{\left(m\right)}(\tau ) & 0\\ \vdots & \vdots & \ddots & \vdots & \ddots & \vdots & \vdots & \vdots \\ W_{l}^{\left(1\right)}(\tau ) & W_{l}^{\left(2\right)}(\tau ) & \cdots & W_{l}^{\left(u\right)}(\tau ) & \cdots & W_{l}^{\left(m-1\right)}(\tau ) & W_{l}^{\left(m\right)}(\tau ) & 0\\ \vdots & \vdots & \ddots & \vdots & \ddots & \vdots & \vdots & \vdots \\ W_{m-1}^{\left(1\right)}(\tau ) & W_{m-1}^{\left(2\right)}(\tau ) & \cdots & W_{m-1}^{\left(u\right)}(\tau ) & \cdots & W_{m-1}^{\left(m-1\right)}(\tau ) & W_{m-1}^{\left(m\right)}(\tau ) & 0\\ W_{m}^{\left(1\right)}(\tau ) & W_{m}^{\left(2\right)}(\tau ) & \cdots & W_{m}^{\left(u\right)}(\tau ) & \cdots & W_{m}^{\left(m-1\right)}(\tau ) & W_{m}^{\left(m\right)}(\tau ) & 0\\ 0 & 0 & \cdots & 0 & \cdots & 0 & 0 & C \end{array}\right],\;\gamma (\tau )=\left[\begin{array}{c} \gamma^{\left(1\right)}(\tau )\\ \gamma^{\left(2\right)}(\tau )\\ \vdots \\ \gamma^{\left(l\right)}(\tau )\\ \vdots \\ \gamma^{\left(m-1\right)}(\tau )\\ \gamma^{\left(m\right)}(\tau ) \end{array}\right]$$

then, Equation (7) has the following linearized form:

(18) A(τ+1)=W(τ+1,τ)A(τ)+γ(τ)

where γ(k) is the modeling error.

In the same way, the linear matrix form of the measurement model can be obtained:

(19) Γ(τ+1)=χ(τ+1,τ)A(τ+1)+θ(τ+1)

where $\chi (\tau +1,\tau )=\left[\begin{array}{ccccccccc} \chi_{1}^{\left(1\right)}(\tau +1) & \chi_{1}^{\left(2\right)}(\tau +1) & \cdots & \chi_{1}^{\left(u\right)}(\tau +1) & \cdots & \chi_{1}^{\left(m-1\right)}(\tau +1) & \chi_{1}^{\left(m\right)}(\tau +1) & 0 & 0\\ \chi_{2}^{\left(1\right)}(\tau +1) & \chi_{2}^{\left(2\right)}(\tau +1) & \cdots & \chi_{2}^{\left(u\right)}(\tau +1) & \cdots & \chi_{2}^{\left(m-1\right)}(\tau +1) & \chi_{2}^{\left(m\right)}(\tau +1) & 0 & 0 \end{array}\right]$ , $\Gamma (\tau +1)=\left[\begin{array}{c} \Gamma_{1}(\tau )\\ \Gamma_{2}(\tau ) \end{array}\right]$ , and $\theta (\tau +1)=\left[\begin{array}{c} \theta_{1}(\tau +1)\\ \theta_{2}(\tau +1) \end{array}\right]$ is the modeling error.

### 4.3. Design of Higher Order Extended Kalman Filter

For linear models, KF-based filters are given. Given the initial value A(0) , when γ(τ) and θ(τ+1) are Gaussian white noise with zero mean, the variances are recorded as ϑ and η , respectively.

A recursive filter can be designed as follows:

(20) $$\hat{A}(\tau +1|\tau )=W(\tau +1,\tau )\hat{A}(\tau |\tau )$$

(21) $$\lambda (\tau +1|\tau )=W(\tau +1,\tau )\lambda (\tau |\tau )W^{T}(\tau +1,\tau )+\vartheta (\tau )$$

(22) $$Κ(\tau +1)=(\lambda (\tau +1|\tau )\chi^{T}(\tau +1)){\left(\chi (\tau +1)\lambda (\tau +1|\tau )\chi^{T}(\tau +1)+\eta (\tau +1)\right)}^{-1}$$

(23) $$\hat{A}(\tau +1|\tau +1)=\hat{A}(\tau +1|\tau )+Κ(\tau +1)(\Gamma (\tau +1)-\chi (\tau +1)\hat{A}(\tau +1|\tau ))$$

(24) λ(τ+1|τ+1)=(I−Κ(τ+1)χ(τ+1))λ(τ+1|τ)

## 5. Higher Order Extended Kalman Filter Design Based on Maximum Correlation Entropy

### 5.1. Non-Gaussian Modeling of State Vector Based on Multivariate Information Observation

System status A(τ) estimates $\hat{A}(\tau |\tau )$ and estimated error covariance λ(τ|τ) are obtained based on Κ [19]. The filtering equation predicts a step prediction estimation value $\tilde{A}(\tau +1|\tau )$ and corresponding step prediction error covariance matrix λ(k+1|k) .

The step prediction estimate error of system status A(τ+1) is as follows:

(25) $$\begin{array}{l} \tilde{A}(\tau +1|\tau )=A(\tau +1)-\hat{A}(\tau +1|\tau )\\ \;\;\;\;\;\;\;\;\;\;\;\;\;\;\;=W(\tau +1,\tau )\tilde{A}(\tau |\tau )+\gamma (\tau ) \end{array}$$

and it can be modified into a measurement model for system status A(τ+1) as follows:

(26) $$\hat{A}(\tau +1|\tau )=A(\tau +1)-\tilde{A}(\tau +1|\tau )$$

where $\hat{A}(\tau +1|\tau )$ is a measurement of system status A(τ+1) , while $\tilde{A}(\tau +1|\tau )$ is the measurement error. Finally, the combined measurement model is as follows:

(27) $$\left[\begin{array}{c} \hat{A}(\tau +1|\tau )\\ \Gamma (\tau +1) \end{array}\right]=\left[\begin{array}{c} I\\ \chi (\tau +1) \end{array}\right]A(\tau +1)+\varpi^{\left(j\right)}(\tau +1)$$

where *I* is a unit array for the corresponding dimension, $\varpi^{\left(j\right)}(\tau +1)=\left[\begin{array}{c} -\tilde{A}(\tau +1|\tau ))\\ \theta^{\left(j\right)}(\tau +1) \end{array}\right]$ , and

(28) $$E\left[\varpi^{\left(i\right)}(\tau +1){\left(\varpi^{\left(i\right)}\right)}^{T}(\tau +1)\right]=\left[\begin{array}{cc} \tilde{\lambda }(\tau +1|\tau ) & 0\\ 0 & \tilde{\eta }(\tau +1) \end{array}\right]$$

According to Equation (20), a step prediction error covariance of the system state λ(τ+1) is received as follows:

(29) $$\tilde{\lambda }(\tau +1|\tau )=W(\tau +1,\tau )\lambda (\tau |\tau )W^{T}(\tau +1,\tau )+\vartheta (\tau )$$

where $\vartheta (\tau )=diag\{\vartheta^{\left(1\right)}(\tau ),\vartheta^{\left(2\right)}(\tau ),\cdots ,\vartheta^{\left(m\right)}(\tau )\}$ and the $\vartheta^{\left(2\right)}(\tau ),\cdots ,\vartheta^{\left(m\right)}(\tau )$ is a covariance matrix of random error vectors $\gamma^{\left(2\right)}(\tau ),\cdots ,\gamma^{\left(r\right)}(\tau )$ when the higher order hidden variable $\alpha^{\left(2\right)}(\tau ),\cdots ,\alpha^{\left(m\right)}(\tau )$ is dynamically modeled. $\vartheta^{\left(1\right)}(\tau )$ is the original system status model (Equation (16)) of the non-Gaussian model error $w^{\left(1\right)}(k)$ , and calculates the second-order statistic after obtaining a limited number of samples:

(30) $${\tilde{\vartheta }}^{\left(1\right)}(\tau )=\frac{1}{N}\sum_{j=1}^{N}\left\{[\gamma^{\left(1,j\right)}(\tau )-\bar{\gamma }(\tau )]{\left[\gamma^{\left(1,j\right)}(\tau )-\bar{\gamma }(\tau )\right]}^{T}\right\}$$

In Equation (23), $\tilde{\eta }(\tau +1)$ is the calculated second-order statistic calculated after the non-Gaussian model error θ(τ+1) in the original system measurement state model (Equation (8)), obtaining a limited sample:

(31) $$\tilde{\eta }(\tau +1)=\frac{1}{N}\sum_{j=1}^{N}\left\{[\theta^{\left(j\right)}(\tau +1)-\bar{\theta }(\tau +1)]{\left[\theta^{\left(j\right)}(\tau +1)-\bar{\theta }(\tau +1)\right]}^{T}\right\}$$

where $\theta^{\left(j\right)}(\tau +1)$ is the jth realization vector of the non-Gaussian random noise vector θ(τ+1) .

### 5.2. The Statistical Independence Process of Each Component in the Non-Gaussian Modeling Error Vector ϖ(τ+1) in the Comprehensive Measurement Model

The vector ϖ(τ+1) in the comprehensive measurement model Equation (22) is a non-Gaussian modeling error vector, and its components are not statistically independent. In order to use the correlation entropy form of the multidimensional independent vector shown in Equation (19), the one-dimensional non-Gaussian vector ϖ(τ+1) needs to be transformed into statistical independence.

From $\lambda (\tau +1|\tau )=E\left\{\left[A(\tau +1|\tau )-A(\tau |\tau )\right]{\left[A(\tau +1|\tau )-A(\tau |\tau )\right]}^{T}\right\}$ , λ(τ+1|τ) is a positive definite matrix. Similarly, in Equation (26), $\tilde{\eta }(\tau +1)$ is also a positive definite matrix. For this reason, Equation (23) is further expressed as follows:

(32) $$\begin{array}{l} E\left\{\varpi^{\left(i\right)}(\tau +1){\left(\varpi^{\left(i\right)}\right)}^{T}(\tau +1)\right\}=\left[\begin{array}{cc} \Lambda_{\alpha }(\tau +1|\tau )\Lambda_{\alpha }^{T}(\tau +1|\tau ) & 0\\ 0 & \Lambda_{\Gamma }(\tau +1)\Lambda_{\Gamma }^{T}(\tau +1) \end{array}\right]\\ \;\;\;\;\;\;\;\;\;\;\;\;\;\;\;\;\;\;\;\;\;\;\;\;\;\;\;\;\;\;\;\;\;\;\;\;\;\;=\Lambda (\tau +1)\Lambda^{T}(\tau +1) \end{array}$$

where $\Lambda_{\alpha }(\tau +1)$ and $\Lambda_{\Gamma }(\tau +1)$ are the Cholesky factor matrices of $\tilde{\lambda }(\tau +1|\tau )$ and $\tilde{\eta }(\tau +1)$ , respectively.

Applying $\Lambda^{-1}(\tau +1)$ to both sides of Equation (22), respectively, yields:

(33) $$\Lambda^{-1}(\tau +1)\left[\begin{array}{c} \hat{A}(\tau +1|\tau )\\ \Gamma (\tau +1) \end{array}\right]=\Lambda^{-1}(\tau +1)\left[\begin{array}{c} I\\ \chi (\tau +1) \end{array}\right]A(\tau +1)+\Lambda^{-1}(\tau +1)\varpi^{\left(j\right)}(\tau +1)$$

where

$$D(\tau +1)=\Lambda^{-1}(\tau +1)\left[\begin{array}{c} \hat{A}(\tau +1|\tau )\\ T(\tau +1) \end{array}\right],S(\tau +1)=\Lambda^{-1}(\tau +1)\left[\begin{array}{c} I\\ \chi (\tau +1) \end{array}\right],e(\tau +1)=\Lambda^{-1}(\tau +1)\varpi (\tau +1)$$

The above equation can be further simplified as follows:

(34) D(τ+1)=S(τ+1)A(τ+1)+e(τ+1)

because

(35) $$\begin{array}{l} E\left\{e(\tau )e^{T}(\tau )\right\}=E\left\{\left[\Lambda^{-1}(\tau +1)\varpi (\tau +1)\right]{\left[\Lambda^{-1}(\tau +1)\varpi (\tau +1)\right]}^{T}\right\}\\ \;\;\;\;\;\;\;\;\;\;\;\;\;\;\;\;\;\;\;\;\;=\Lambda^{-1}(\tau +1)E\left\{\varpi (\tau +1)\varpi^{T}(\tau +1)\right\}{\left(\Lambda^{-1}\left(\tau +1\right)\right)}^{T}\\ \;\;\;\;\;\;\;\;\;\;\;\;\;\;\;\;\;\;\;\;\;=\Lambda^{-1}(\tau +1)\Lambda (\tau +1)\Lambda^{T}(\tau +1){\left(\Lambda^{-1}\left(\tau +1\right)\right)}^{T}\\ \;\;\;\;\;\;\;\;\;\;\;\;\;\;\;\;\;\;\;\;\;=I \end{array}$$

Therefore, after the non-Gaussian modeling error random variable ϖ(τ+1) undergoes the equivalent transformation of the matrix $\Lambda^{-1}(\tau +1)$ , the components of the random e(τ+1) are statistically independent.

### 5.3. Implementation Process of a Higher Order Extended Kalman Filter Based on Maximum Entropy

The filtering process of the extended Kalman filter (H-MCEKF) based on the maximum correlation entropy is as follows (see [20] for the specific derivation process):

1. The filter initialization obtains the initial filter value $\hat{A}(0)$ and the covariance λ(0) , choosing a suitable core bandwidth ο and a small positive number ε ;
2. Taylor networks are used for system identification to obtain the parameters in the equations, using the expanded item and the remainder as the new hidden variables. A pseudolinearization process is performed to obtain the pseudolinear form of the system;
3. Equations (20) and (21) are used to obtain $\hat{X}(k+1|k)$ and P(k+1|k) , respectively, while Cholesky decomposition is used to obtain $B_{p}(k+1|k)$ ;
4.
t=1 and $\hat{A}{\left(\tau +1|\tau +1\right)}_{0}=\hat{A}(\tau +1|\tau )$ are taken, where $\hat{A}{\left(\tau +1|\tau +1\right)}_{t}$ represents the estimated state of the fixed-point iteration *t*;
5. The starting fixed-point iterative algorithm is as follows:
(36) $${\tilde{e}}_{i}\left(\tau +1\right)=d_{i}\left(\tau +1\right)-s_{i}\left(\tau +1\right)\hat{A}{\left(\tau +1|\tau +1\right)}_{t-1}$$
  where $e_{i}$ is the ith element of e :
(37) $${\tilde{C}}_{\alpha }(\tau +1)=diag\left(G_{ο}\left({\tilde{e}}_{1}(\tau +1)\right),\cdots ,G_{ο}\left({\tilde{e}}_{m}(\tau +1)\right)\right)$$
(38) $${\tilde{C}}_{\Gamma }(\tau +1)=diag\left(G_{ο}\left({\tilde{e}}_{r+1}(\tau +1)\right),\cdots ,G_{ο}\left({\tilde{e}}_{m+m}(\tau +1)\right)\right)$$
(39) $$\tilde{H}(\tau +1)=\Lambda_{\Gamma }(\tau +1){\tilde{C}}_{\Gamma }^{-1}(\tau +1)\Lambda_{\Gamma }^{T}(\tau +1)$$
(40) $$\tilde{\lambda }(\tau +1|\tau )=\Lambda_{\alpha }(\tau +1|\tau ){\tilde{C}}_{\alpha }^{-1}(\tau +1)\Lambda_{\alpha }{}^{T}(\tau +1|\tau )$$
(41) $$\tilde{Κ}(\tau +1)=\tilde{\lambda }(\tau +1|\tau )\chi^{T}{\left(\chi \tilde{\lambda }(\tau +1|\tau )\chi^{T}+\tilde{H}(\tau +1)\right)}^{-1}$$
(42) $$\hat{A}{\left(\tau +1|\tau +1\right)}_{t}=\hat{A}(\tau +1|\tau )+\tilde{Κ}(\tau +1)(\Gamma (\tau +1)-H\hat{A}(\tau +1|\tau ))$$
  The estimates of the current iteration step are compared with those of the previous iteration and, if satisfied,
(43) $$\frac{\left\|\hat{A}{\left(\tau +1|\tau +1\right)}_{t}-\hat{A}{\left(\tau +1|\tau +1\right)}_{t-1}\right\|}{\left\|\hat{A}{\left(\tau +1|\tau +1\right)}_{t-1}\right\|}\leq \varepsilon$$
  then $\hat{A}(\tau +1|\tau +1)=\hat{A}{\left(\tau +1|\tau +1\right)}_{t},\lambda (\tau +1|\tau +1)=\tilde{\lambda }(\tau +1|\tau )$ , and the value of the pseudovariable can be updated, or the iteration can be repeated;
6.
τ=τ+1 , and steps (3–5) are repeated until the end of filtering.

## 6. Simulated Cases

This section verifies the validity of the proposed method by providing two cases: one in which the state equation is a nonlinear equation and the measurement equation is a linear equation, and one in which the state and measurement equations are both nonlinear.

### 6.1. Case 1

Consider a nonlinear system in which the state equation is a nonlinear model and the measurement equation is a linear model:

$$\begin{array}{l} \{\begin{array}{l} x_{1}(k+1)=\left(0.8-0.5e^{-x_{1}^{2}(k)\left(1+e^{-0.015k}\right)}\right)x_{1}(k)-\left(0.3+0.9e^{-x_{1}{}^{2}(k)\left(1+0.5\sin\left(\frac{\pi }{2}k\right)\right)}\right)x_{2}(k)+w_{1}(k)\\ x_{2}(k+1)=1.2\left(1-e^{-0.8k}\right)x_{2}(k)+0.11x_{1}(k)+\cos\left(1+x_{2}^{2}(k)\right)+e^{-0.8k}x_{1}^{4}(k)+w_{2}(k) \end{array}\\ \{\begin{array}{c} y_{1}(k+1)=x_{1}(k+1)+v_{1}(k+1)\\ y_{2}(k+1)=x_{2}(k+1)+v_{2}(k+1) \end{array} \end{array}$$

where the initial value x(0) is a random value of [0,1] , the initial estimation error covariance P(0|0)=0.1×diag(1,1) , and the process noise and measurement noise have the following characteristics:

$$\begin{array}{c} w_{1}(k)\sim 0.9N(0,0.01)+0.1N(0,0.2),w_{2}(k)\sim 0.9N(0,0.02)+0.1N(0,0.2)\\ v_{1}(k)\sim 0.9N(0,0.01)+0.1N(0,2),v_{2}(k)\sim 0.9N(0,0.02)+0.1N(0,2) \end{array}$$

**Figure 2:**
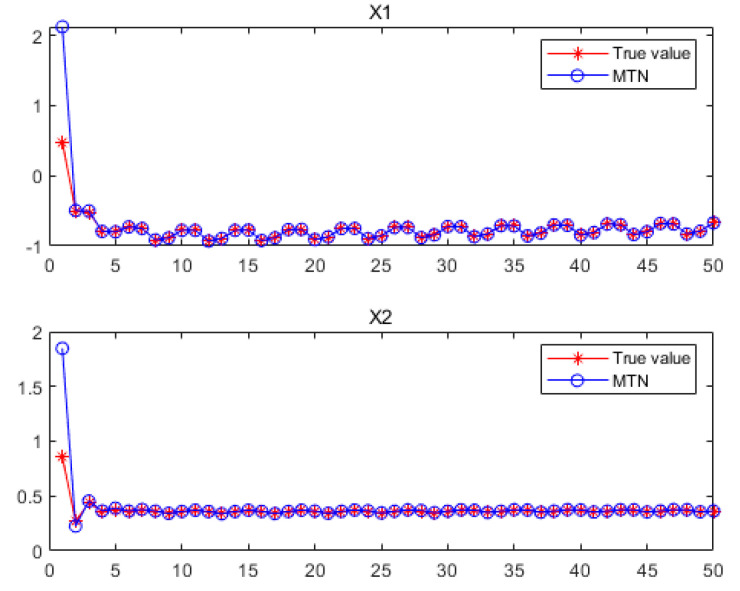
Graph of the MTN identification system in case 1.

**Figure 3:**
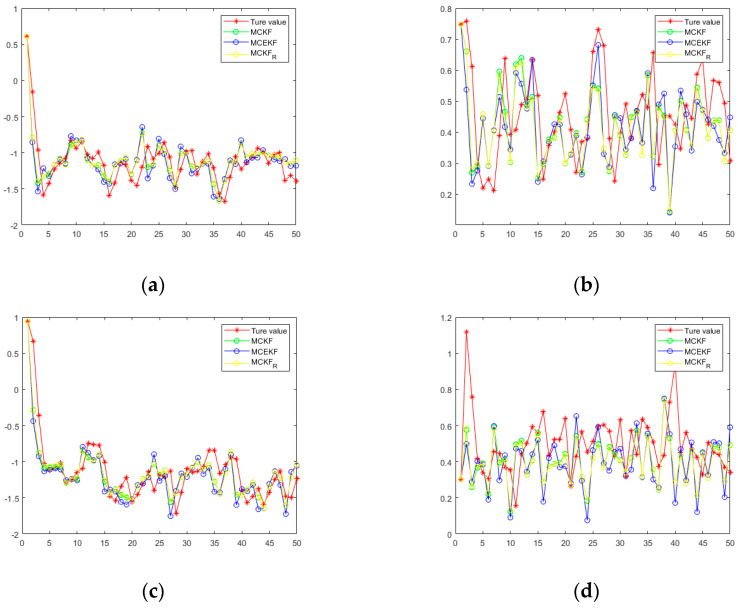
(**a**,**b**) Set parameters: $\sigma =5,\varepsilon =10^{-6}$ in case 1; (**c**,**d**) set parameters: $\sigma =10,\varepsilon =10^{-6}$ in case 1.

**Figure 4:**
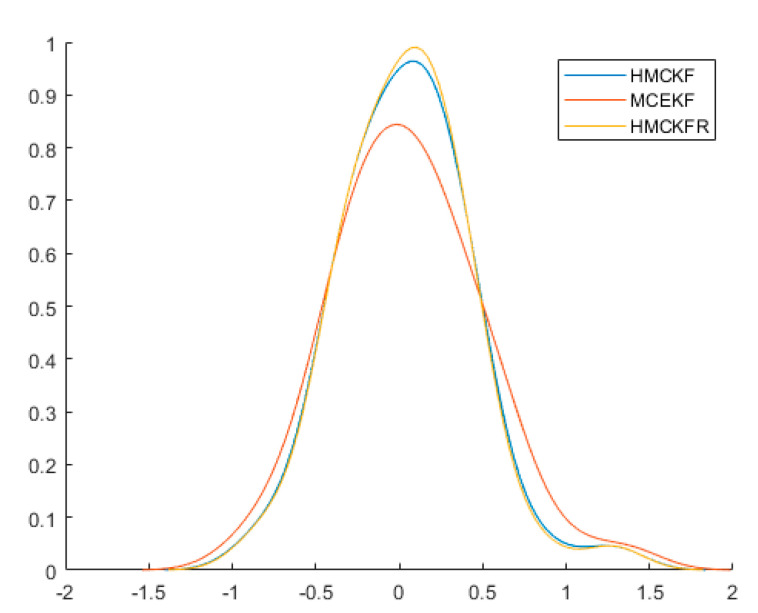
Probability densities of $x_{1}$ estimation errors with the three filters in case 1.

**Figure 5:**
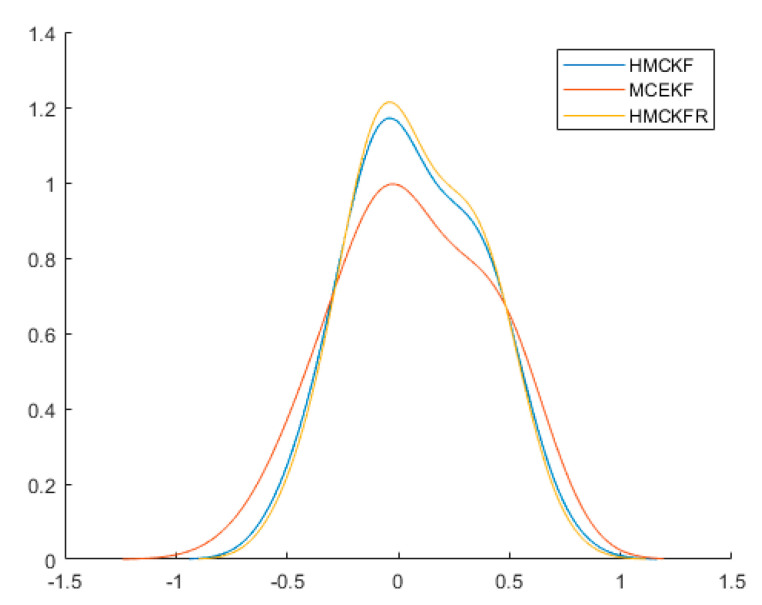
Probability densities of $x_{2}$ estimation errors with the three filters in case 1.

Figure 2 shows a diagram of the MTN identification system, while Figure 3 shows the estimated values of state variables $x_{1}$ and $x_{2}$ under the three filtering methods. From [21], we know the influence of ε is not significant compared with the kernel bandwidth σ. The parameters are set at $\varepsilon =10^{-6}$ . Table 1 and Table 2 show the mean squared error and the mean relative error, respectively, of the estimated values under the three algorithms, which are computed as averages over 100 independent Monte Carlo runs, with each run containing 50 time steps. When σ=5 , the three algorithms all obtain better filtering results. Figure 4 and Figure 5 show the probability densities of the estimation errors when estimating the states $x_{1}$ and $x_{2}$ , respectively, when the parameters are $\varepsilon =10^{-6}$ and σ=5 . All of the results confirm that the proposed H-MCKF (design method for a higher order extended Kalman filter based on maximum correlation entropy and a Taylor network system) can outperform the MCEKF (maximum correntropy extended Kalman filter) significantly when the system is disturbed by non-Gaussian processes and measurement noise, and the H-MCKF_R (H-MCKF with the remainder of the state equation) further improves the filtering performance of the H-MCKF.

### 6.2. Case 2

Consider a nonlinear system in which the state equation and the measurement equation are both nonlinear models:

$$\begin{array}{l} \{\begin{array}{l} x_{1}(k+1)=\cos\left(0.5x_{1}(k)+\frac{2.5x_{2}\left(k\right)}{1+x_{1}{}^{2}(k)+8\cos(1.2k)}\right){+w}_{1}(k)\\ x_{2}(k+1)=\sin(x_{1}{}^{2}{(k))+w}_{2}(k) \end{array}\\ \{\begin{array}{c} y_{1}(k)=\cos(x_{1}\left(k\right)+\sin(x_{1}{}^{3}\left(k\right)))+v_{1}(k+1)\\ y_{2}(k)=\sin(x_{2}(k)-\sin(x_{2}{}^{3}\left(k\right)))+v_{2}(k+1) \end{array} \end{array}$$

where the initial value x(0) is a random value of [0, 1], the initial estimation error covariance P(0|0)=0.1×diag(1,1) , and the process noise and measurement noise have the following characteristics:

$$\begin{array}{c} w_{1}(k)\sim 0.9N(0,0.01)+0.1N(0,0.2),w_{2}(k)\sim 0.9N(0,0.02)+0.1N(0,0.2)\\ v_{1}(k)\sim 0.9N(0,0.01)+0.1N(0,2),v_{2}(k)\sim 0.9N(0,0.02)+0.1N(0,2) \end{array}$$

**Figure 6:**
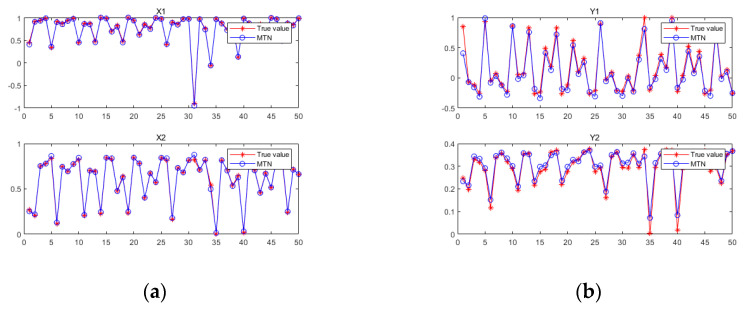
(**a**) Graph of the MTN identify the state equation in case 2. (**b**) Graph of the MTN identify the measurement equation in case 2.

**Figure 7:**
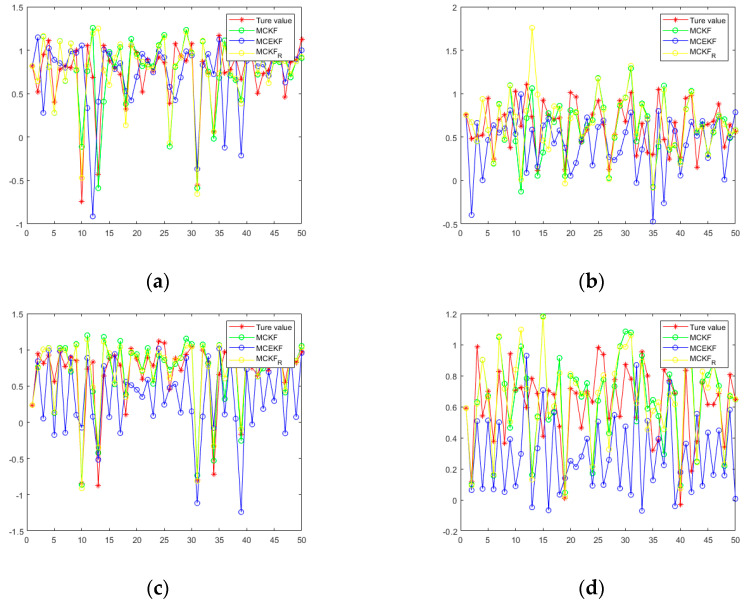
(**a**,**b**) Set parameters: $\sigma =5,\;\varepsilon =10^{-6}$ in case2; (**c**,**d**) set parameters: $\sigma =10,\;\varepsilon =10^{-6}$ in case 2.

**Figure 8:**
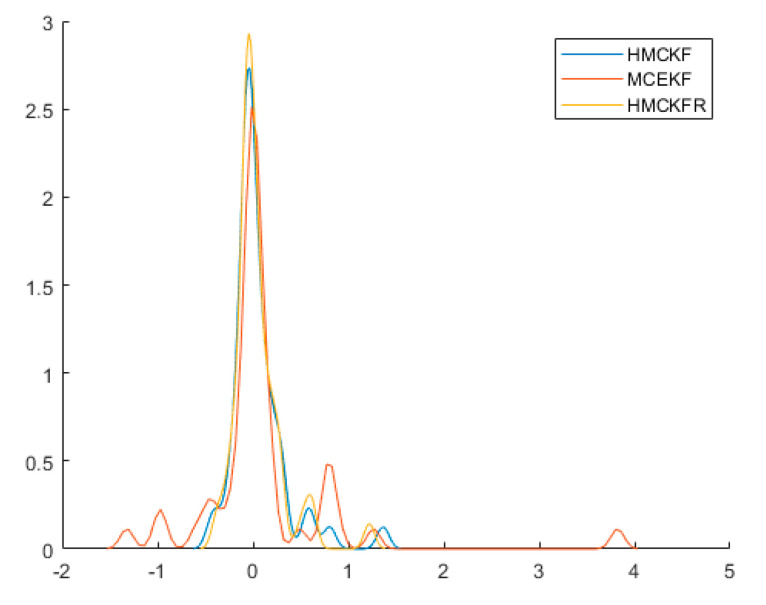
Probability densities of $x_{1}$ estimation errors with the three filters in case 2.

**Figure 9:**
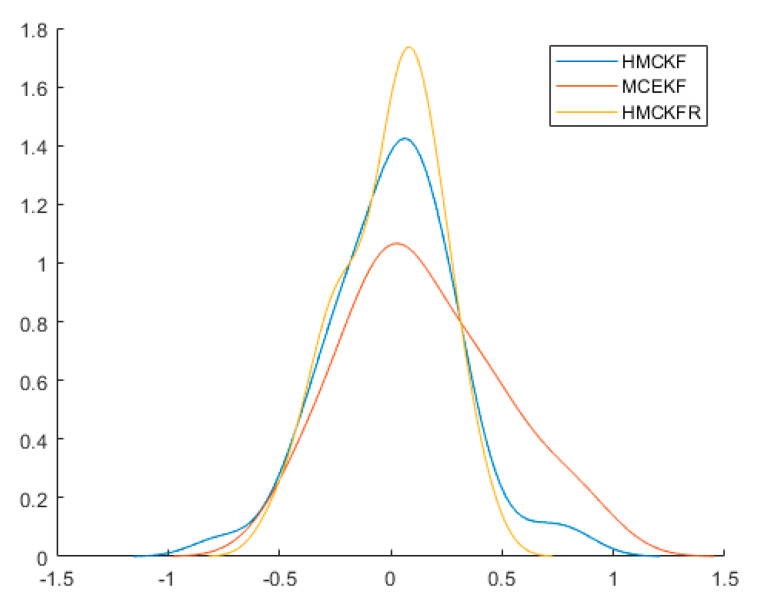
Probability densities of $x_{2}$ estimation errors with the three filters in case 2.

Figure 6 shows a diagram of the MTN identification system, while Figure 7 shows the estimated values of state variables $x_{1}$ and $x_{2}$ under the three filtering methods. Similar to case 1, the parameters are set at $\varepsilon =10^{-6}$ . Table 3 and Table 4 show the mean squared error and the mean relative error, respectively, of the estimated values under the three algorithms, which are computed as averages over 100 independent Monte Carlo runs, with each run containing 50 time steps. When σ=5 , the three algorithms all obtain better filtering results. Figure 8 and Figure 9 show the probability densities of the estimation errors when estimating the states $x_{1}$ and $x_{2}$ , respectively, when the parameters are $\varepsilon =10^{-6}$ and σ=5 . All of the results confirm that the proposed H-MCKF can outperform the MCKF significantly when the system is disturbed by non-Gaussian processes and measurement noise, and the H-MCKF_R further improves the filtering performance of the H-MCKF when the state and measurement equations are both nonlinear.

## 7. Conclusions

This paper considered a wide range of filter design problems for the state estimation of multivariable dynamic systems, which consist of a strong nonlinear dynamic model and a strong nonlinear observation model. Firstly, we transformed those strong nonlinear models into a higher order polynomial series using a multidimensional Taylor network. Secondly, all higher order items in the polynomial series were defined as hidden variables. Those higher order series were then rewritten as their pseudolinear equivalents. Thirdly, dynamic relationships between all hidden variables and known variables were constructed using the multidimensional Taylor network. Combining the original model of pseudolinearization with the higher order hidden variable dynamic model, linear dynamic models fitted to a standard Kalman filter were presented. Finally, considering that a finite number of samples from modeling error can be obtained, we built the higher order extended Kalman filter based on maximum correlation entropy, and acquired better filter performance than offered by the existing MCEKF [22].

Outlook: There exist several challenges worthy of further research. Firstly, the proposed higher order extended Kalman filter based on maximum correlation entropy is an online iteration process that obtains state estimation constantly, but, as such, it loses one important function possessed by the standard Kalman filter: the ability to operate in real time. Secondly, the linearized model parameters of the original nonlinear model and the hidden variable dynamic model were identified by local time period data; thus, they need to be updated with new time period data in order to fit the time dynamics of the system. Thirdly, in this paper, on the basis of defining all of the hidden variables, we established a linear form of the strong nonlinear model in an expanded state with the original variables and all hidden variables, and obtained better estimation performance than that of a standard EKF; if measurements can be expanded in the same manner as state, we believe that such a filter may offer better estimation performance than the one established by this paper.

## Figures and Tables

**Table 1:** The mean squared error using the three methods in case 1.

		$\mathrm{MSE}\;\mathrm{of}\;x_{1}$			$\mathrm{MSE}\;\mathrm{of}\;x_{2}$		
σ	ε	MCEKF	H-MCKF	H-MCKF_R	MCEKF	H-MCKF	H-MCKF_R
σ=2	$\varepsilon =10^{-6}$	0.2073	0.1815	0.1694	0.1000	0.0774	0.0738
σ=5	$\varepsilon =10^{-6}$	0.1974	0.1244	0.1225	0.1100	0.0964	0.0921
σ=10	$\varepsilon =10^{-6}$	0.2282	0.1669	0.1636	0.1158	0.0925	0.0888
σ=20	$\varepsilon =10^{-6}$	0.2244	0.1602	0.1572	0.1160	0.0916	0.0880

**Table 2:** The mean relative error using the three methods in case 1.

		$\mathrm{MRE}\;\mathrm{of}\;x_{1}$			$\mathrm{MRE}\;\mathrm{of}\;x_{2}$		
σ	ε	MCEKF	H-MCKF	H-MCKF_R	MCEKF	H-MCKF	H-MCKF_R
σ=2	$\varepsilon =10^{-6}$	0.3372	0.2405	0.2403	0.2354	0.2202	0.2084
σ=5	$\varepsilon =10^{-6}$	0.3462	0.2953	0.2906	0.2679	0.2485	0.2448
σ=10	$\varepsilon =10^{-6}$	0.3658	0.3052	0.2986	0.2745	0.2469	0.2426
σ=20	$\varepsilon =10^{-6}$	0.3634	0.3009	0.2945	0.2753	0.2466	0.2419

**Table 3:** The mean squared error using the three methods in case 2.

		$\mathrm{MSE}\;\mathrm{of}\;x_{1}$			$\mathrm{MSE}\;\mathrm{of}\;x_{2}$		
σ	ε	MCEKF	H-MCKF	H-MCKF_R	MCEKF	H-MCKF	H-MCKF_R
σ=2	$\varepsilon =10^{-6}$	0.4017	0.1230	0.1219	0.2090	0.0907	0.0883
σ=5	$\varepsilon =10^{-6}$	0.1148	0.1241	0.1233	0.2542	0.1200	0.1183
σ=10	$\varepsilon =10^{-6}$	0.3221	0.1254	0.1248	0.2220	0.1207	0.1193
σ=20	$\varepsilon =10^{-6}$	0.4040	0.1257	0.1251	0.2218	0.1208	0.1196

**Table 4:** The mean relative error using the three methods in case 2.

		$\mathrm{MRE}\;\mathrm{of}\;x_{1}$			$\mathrm{MRE}\;\mathrm{of}\;x_{2}$		
σ	ε	MCEKF	H-MCKF	H-MCKF_R	MCEKF	H-MCKF	H-MCKF_R
σ=2	$\varepsilon =10^{-6}$	0.5106	0.2337	0.2306	0.3742	0.2355	0.2316
σ=5	$\varepsilon =10^{-6}$	0.2147	0.2551	0.2530	0.3070	0.2652	0.2645
σ=10	$\varepsilon =10^{-6}$	0.4527	0.2570	0.2553	0.3824	0.2661	0.2659
σ=20	$\varepsilon =10^{-6}$	0.4764	0.2575	0.2558	0.3789	0.2663	0.2662
